# Vitamin D status in the adult population of Bursa-Turkey

**DOI:** 10.1080/13814788.2020.1846712

**Published:** 2020-12-09

**Authors:** Olgun Göktaş, Canan Ersoy, Ilker Ercan, Fatma Ezgi Can

**Affiliations:** a Family Practice Unit, Uludağ University Family Health Center, Bursa, Turkey; b Department of Internal Medicine, Division of Endocrinology and Metabolism, Uludağ University Faculty of Medicine, Bursa, Turkey; c Department of Biostatistics, Uludağ University Faculty of Medicine, Bursa, Turkey; d Department of Biostatistics, İzmir Katip Çelebi University Faculty of Medicine, İzmir, Turkey

**Keywords:** General practice/family medicine, vitamin D, seasonal variation, gender

## Abstract

**Background:**

Vitamin D, along with parathyroid hormone (PTH) and calcitonin, is an important hormone that affects bone and calcium metabolism.

**Objectives:**

To evaluate the vitamin D status and its seasonal variation in the adult population of Bursa.

**Methods:**

Since there is not enough information about vitamin D levels, 25 OH vitamin D levels were analysed retrospectively from the records of 11,734 adult subjects (9142 women, 2592 men) admitted to 24 family health centres located in different districts of Bursa between 1 December 2017 and 30 November 2018. Some tests that can affect vitamin D levels, demographic features, and accompanying comorbidities were also evaluated. A face-to-face questionnaire was administered to subjects who were willing to answer (*n* = 2965).

**Results:**

The mean serum vitamin D level was 16.6 ± 11.5 ng/mL in the entire population, 15.8 ± 11.7 ng/mL in women and 19.5 ± 9.9 ng/mL in men. The percentage of subjects with a vitamin D level <20 ng/mL was highest in March–May and lowest in the September–November period (80.7% and 69.9% for women, 77.5% and 32.9% for men, respectively).

**Conclusion:**

Vitamin D levels <20 ng/mL are more prominent in women, and decline in spring, in the adult population of Bursa. These results are meaningful for the population living in Bursa, Turkey and the northern European region.

KEY MESSAGES

- Vitamin D levels were <20 ng/mL in the entire population.
- Vitamin D levels <20 ng/mL are more prominent in women and decline in spring.
- The percentage of vitamin D levels <20 ng/mL was highest in summer and lowest in the autumn.

## Introduction

Vitamin D, along with parathyroid hormone (PTH) and calcitonin, is an important hormone that affects bone and calcium metabolism. It also has extra-skeletal effects on insulin resistance and related disorders, immunity, and cancer. Its deficiency, insufficiency or excess in the body causes problems. Today, with the contribution of many factors, vitamin D deficiency or insufficiency is quite common and has become a significant health problem across the world. Although vitamin D deficiency is common, only those at a high risk of 25 (OH) D deficiency are evaluated, and community screening is not recommended [1–4].

Vitamin D is a steroid hormone, 80–90% produced in the skin from 7 dihydrocholesterol by incident ultraviolet (UV) B. The remaining 10–20% is derived from fatty fish, vegetable sources and foods rich in vitamin D. Vitamin D is a prohormone, activated in the liver and kidney. The angle of the incidence of short-wavelength UVB rays (290–315 nm) on the earth’s surface (Zenith angle), when not obtuse, is effective for the synthesis of vitamin D. Although this leads to less benefiting time in the northern European countries, vitamin D synthesis takes place between May and November in our (Turkey’s) latitude. The beneficial angle of the incidence of UVB is usually between 10:00 am to 03:00 pm. In the summer months, when the whole body is exposed to sunlight, at a minimal erythema dose, it synthesises vitamin D, equivalent to approximately 20,000 international units (IU) of oral dose. The minimal erythema dose is reached in approximately 15 min in people with light skin colour. However, the time to reach the minimal erythema dose is 3–4 times longer in individuals with darker skin colour [1,2,5–7].

Apart from the dark skin colour, people with a high risk of vitamin D deficiency are those who are elderly, obese, on medications that accelerate vitamin D metabolism, suffer from spontaneous fractures without trauma, osteoporosis, osteomalacia, malabsorption syndromes such as coeliac disease, inflammatory bowel disease, chronic kidney failure, chronic liver disease and hyperparathyroidism who have insufficient exposure to the sun [1–5,7].

Vitamin D status is determined according to 25 hydroxy (OH) vitamin D levels measured in serum. Vitamin D level may be sufficient (>30 ng/mL), insufficient (20–30 ng/mL), deficient (<20 ng/L) or seriously deficient (<10 ng/mL) [2,4,7].

Although a severe vitamin D deficiency, namely osteomalacia, is rarely seen, vitamin D deficiencies are quite common. The frequency of such deficiencies reported in adults who are 20 years of age and older is approximately 41.6% [8–10]. In different studies conducted in Turkey, the frequency of vitamin D deficiency in adults has been reported to be between 33% and 75% [4,11,12]. These deficiency rates are more critical for Turkish societies living in northern Europe for a long time.

In this study, we aimed to retrospectively evaluate the vitamin D status and its seasonal variation in the adult population of Bursa province, 63.4% of whom live in urban areas and 34.7% in rural areas, with a latitude of approximately 40^°^ located in the Southern Marmara region of western Turkey in the Northern hemisphere.

## Methods

### Study design

After obtaining the necessary approvals, data on 25 OH vitamin D levels measured in patients admitted between 1 December 2017 and 30 November 2018 were analysed retrospectively from the records of adult patients aged ≥18 years who were admitted to 24 family medicine centres located in different districts of Bursa. A questionnaire when they apply to a family doctor for any reason was directed to subjects who were willing to answer (*n* = 2965, 25.3% of those who participated in the study, 61.2% of whom were women and 38.8% were men) concerning lifestyle and dietary habits related to vitamin D.

The results were obtained from a single centralised laboratory. The method used for the measurement of 25 OH vitamin D was a new technology ECLIA (Electro-ChemiLuminescence Immunoassay) with the Roche Cobas E170 biochemistry analyser system.

Besides serum 25 OH vitamin D levels, liver and renal function tests, glycaemic and lipid parameters, C-reactive protein (CRP) levels, demographic features, and accompanying comorbidities were obtained retrospectively from the patients’ records.

### Ethics

The study was performed after the approval of the Health Directorate of Bursa of the Republic of Turkey Ministry of Health (Ref. 60429939/604.02) and the Clinical Ethics Committee of Bursa Uludağ University (Ref. 2017-19/14) and following the Declaration of Helsinki.

### Statistical analysis

The consistency of continuous variables to normal distribution was tested using the Shapiro–Wilk test. Descriptive statistics are given as median (min–max) for continuous variables and n (%) for categorical variables. Kruskal–Wallis, Mann–Whitney *U*, Pearson chi-square and Fisher exact tests were used for comparisons between groups. Bonferroni correction was applied for binary group comparisons. Factors affecting low vitamin D levels were investigated using stepwise logistic regression analysis. Statistical analyses were performed using the IBM SPSS v.21 program. *p* < 0.05 was considered statistically significant.

## Results

Vitamin D levels of 11,893 adult patients [9268 (77.9%) women, and 2625 (22%) men] were obtained. The data of 11,734 patients [9142 (77.9%) women, and 2592 (22%) men] whose vitamin D levels were studied at their first visit to a family doctor for any reason, and who did not receive any vitamin D treatment at the time of their visits were included in the study. A total of 2965 people participated in the questionnaire (*n* = 2965, 25.3% of those who participated in the study, 61.2% of whom were women, and 38.8% were men).

### Population characteristics and seasonal variation

The average age of the 11,734 patients was 46.5 ± 16.9 years; the average age of women was 45.3 ± 16.4 years, and the average age of men was 51.1 ± 17.9 years. The mean serum vitamin D level in the entire population was calculated as 16.6 ± 11.5 ng/mL, which was 15.8 ± 11.7 ng/mL in women and 19.5 ± 9.9 ng/mL in men.

Since seasonal variations affect UVB exposure, vitamin D levels were evaluated in both sexes according to seasons. To determine the season-dependent vitamin D levels, months were grouped as December–February (winter), March–May (spring), June–August (summer) and September–November (autumn). The number of admissions in winter, spring, summer and autumn were 3768 [2995 (79.4%) women, 773 (20.5%) men], 3279 [2533 (77.2%) women, 746 (22.7%) men], 2464 [1911 (77.5%) women, 553 (22.4%) men] and 2223 [1703 (76.6%) women, 520 (23.4%) men], respectively. A statistically significant difference was found in overall vitamin D levels amongst women and men when compared according to season (Table 1). To determine the seasonal variations in vitamin D status, patients in both sexes were divided into two groups with a cut-off level of 20 ng/mL (Table 1).

**Table 1.** Vitamin D values by gender and seasons.

	Vitamin D (ng/mL) (Overall)			Vitamin D categories		
	n	Median (min–max)	*p* value	<20	≥20	*p* value
Women						
December–February (I)	2995	11 (3–70)		2294 (76.6%)	701 (23.4%)	<0.001
March–May (II)	2533	10.2 (3–140)		2044 (80.7%)	476 (19.3%)	
June–August (III)	1911	10.3 (3–140)	<0.001	1541 (80.6%)	357 (19.4%)	
September–November (IV)	1703	14.8 (3–140)		1191 (69.9%)	491 (30.1%)	
All women						
All seasons	9142	12.7 (3–140)		2344 (25.6%)	6798 (74.4%)	
Men						
December–February (I)	773	19 (3–70)		429 (55.5%)	344 (44.5%)	<0.001
March–May (II)	746	14.8 (3–86.9)		578 (77.5%)	163 (22.5%)	
June–August(III)	553	15.4 (3–88)	<0.001	393 (71.1%)	156 (28.9%)	
September–November (IV)	520	23.1 (3–71.8)		171 (32.9%)	339 (67.1%)	
All men						
All seasons	2592	18.2 (3–88)		1110 (42.8%)	1482 (57.2%)	
Total						
All seasons	11,734					

In terms of gender differences, a statistically significant difference was found between the vitamin D levels of men and women in different seasons (*p* < 0.001, *p* < 0.001). In women, no statistical differences were found in vitamin D levels < and ≥20 ng/mL between spring and summer periods. In men, however, statistically significant vitamin D levels < and ≥20 ng/mL were found in all periods.

### Vitamin D status, comorbidity and laboratory parameters

When comorbid situations that might be associated with vitamin D levels were investigated, a statistically significant difference in vitamin D levels was found in patients suffering from type 2 diabetes, hypertension, dyslipidemia and atherosclerotic heart disease. Vitamin D deficiency (<20 ng/mL) appeared to occur less in the above-mentioned comorbid conditions (Table 2).

**Table 2.** Association of comorbidities on vitamin D levels.

Accompanying comorbidity	Vitamin D status				
	n	<20 (ng/mL)	n	≥20 (ng/mL)	*p* value
Liver disease	8280	48 (0.6%)	3454	18 (0.5%)	0.699
Renal disease	8280	133 (1.6%)	3454	65 (1.9%)	0.291
Malabsorbtion	8280	101 (1.2%)	3454	34 (1%)	0.276
Inflammatory Bowel disease	8280	126 (1.5%)	3454	60 (1.7%)	0.395
Obesity	8280	123 (1.5%)	3454	38 (1.1%)	0.102
Type 2 diabetes	8280	197 (2.4%)	3454	113 (3.3%)	0.006
Hypertension	8280	2352 (28.4%)	3454	1250 (36.2%)	<0.001
Dyslipidemia	8280	1082 (13.1%)	3454	601 (17.4%)	<0.001
Atherosclerotic cardiovascular disease	8280	714 (8.6%)	3454	417 (12.1%)	<0.001
Smokers	1014	8 (0.8%)	368	1 (0.3%)	0.459
Fracture					
None (I)		1102 (85.8%)		459 (82.9%)	0.130
Bone Fragility (II)		152 (11.8%)		74 (13.4%)	
Traumatic (III)		30 (2.3%)		21 (3.8%)	

In subjects with lower vitamin D levels, serum creatinine, alanine aminotransferase (ALT), aspartate aminotransferase (AST), total cholesterol, low-density lipoprotein (LDL) cholesterol and insulin values were lower, and phosphorus, calcium and CRP values were higher (Table 3).

**Table 3.** Association of laboratory parameters on vitamin D levels.

Laboratory parameter	Vitamin D status				
	*n*	<20 (ng/mL)	*n*	≥20 (ng/mL)	*p* value
Calcium, Median (min–max)	2861	9.5 (5.2–12.3)	6653	9.4 (6.1–12)	<0.001
Creatinine (mg/dL), Median (min–max)	7703	0.6 (0.1–5.7)	3250	0.7 (0.2–5)	<0.001
ALT (U/L), Median (min–max)	7749	15 (1–411)	3261	16 (1–280)	<0.001
AST (U/L), Median (min–max)	7565	16 (1–393)	3205	17 (7–144)	<0.001
Glucose (mg/dL), Median (min–max)	7931	92 (36–482.5)	3333	92 (43–357)	0.090
HDL cholesterol (mg/dL), Median (min–max)	6840	52 (13–124)	2992	52 (17–145)	0.257
Total cholesterol (mg/dL), Median (min–max)	7372	182 (70–476)	3165	188.5 (74.5–423)	<0.001
LDL cholesterol (mg/dL), Median (min–max)	6723	104 (11–372)	2954	109 (16–312)	<0.001
Triglyceride (mg/dL)	7056	114 (22–1613)	3065	113 (28–810)	0.638
Insulin (mIU/L)	5342	8.7 (0.1–181.5)	2381	7.5 (0.2–146.6)	<0.001
CRP (mg/L), Median (min–max)	6409	0.2 (0–31.5)	2737	0.1 (0–24.1)	<0.001
Phosphorus, Median (min–max)	2425	3.5 (1.8–7.4)	5567	3.5 (1.4–7.3)	0.040

ALT: alanine aminotransferase; AST: aspartate aminotransferase; HDL: high-density lipoprotein; LDL: low-density lipoprotein; CRP: C-reactive protein.

When gender, age, concomitant diseases, annual cigarette pack smoked, daily milk and dairy intake, clothing style and indoor work, sun exposure duration, sunscreen usage, and body mass index variables were considered in a stepwise logistic regression analysis, the levels of vitamin D were found to be affected by only gender and milk and dairy product consumption. The milk and dairy product consumption variables did not show a significant effect. It was observed that vitamin D level in women was 8.4 times lower than in men (Table 4).

**Table 4.** Determination of factors affecting vitamin D level.

	OR	%95 Confidence interval	*p* value
Gender (Ref: Male)	8.4	3.8–18.4	<0.001
Milk (Ref: milk and dairy product consumption)			0.085
Dairy product	2.5	0.5–10.8	0.220
Milk	0.1	0–1.1	0.071
None	0.7	0.2–1.7	0.433

Model significance: *p* < 0.001.

## Discussion

### Main findings

Low vitamin D levels are seen in increasing frequency in Turkey, although it has a sunny climate [10]. Our results indicated that low vitamin D levels are more prominent in women and in spring in the urban population of our city, Bursa. There are a few studies reported from Turkey, mainly conducted on female subjects, and older adults, and from different regions of Turkey [4,11–13].

This is the first population-based study conducted in Bursa with a higher number of subjects (*n* = 11,734; 9142 women and 2592 men) that has the power to represent the population living in Bursa, which was 3,056,120 in the year 2019 [14].

The average age of the 11,734 patients was 46.5 years, within the middle age group being active in life. The mean serum vitamin D level of the entire population was 16.6 ng/mL, which was <20 ng/mL (deficient range) [2,4,7,15,16]. The mean serum vitamin D level in women was lower than that in men (15.8 ng/mL vs. 19.5 ng/mL, respectively). When the serum vitamin D level was evaluated in terms of adequacy, it was found that 70.6% of the general population, 74.4% of women and 57.2% of men had a vitamin D level <20 ng/mL. These values were similar to those reported in other studies [4,17–23].

Seasonal variations are essential for sun exposure and vitamin D levels. In our study, a statistically significant difference was found in women and men when vitamin D values were compared according to season. In both women and men, vitamin D levels were lowest in the March–May period and highest in the September–November period (10.2 ng/mL and 14.8 ng/mL for women, 14.8 ng/mL and 23.1 ng/mL for men, respectively). Similarly, vitamin D levels <20 ng/mL were highest in the March–May period and lowest in the September–November period in both genders (80.7% and 69.9% for women, 77.5% and 32.9% for men, respectively). These results were similar to those of studies conducted in other European countries [17,23–26].

In our study, women had lower levels of vitamin D compared to men in all seasons, consistent with many studies in the literature [4,11,17,27]. In our study group, logistic regression analysis indicated that gender is an essential factor in vitamin D levels, and low vitamin D levels in women were 8.4 times higher than in men. This variation might be due to body fat content and distribution differences among men and women [28] as well as less exposure to sunlight due to clothing habits and less time spent outdoors for women in our study population.

Comorbid conditions like nutrition, smoking and daily life habits may influence vitamin D levels. As a prohormone, vitamin D is activated in the liver and kidneys. Calcium is absorbed through the intestinal system *via* activated vitamin D. Low calcium intake, malabsorption and calcium metabolism disorders may lead to bone fractures. According to some studies, lower vitamin D levels are thought to be related to increased inflammation, hyperinsulinemia, insulin resistance and related disorders such as obesity, hypertension, dyslipidemia and atherosclerotic cardiovascular diseases. However, there are conflicting results [1,2,4–6,29,30].

When the accompanying comorbid situations were investigated in our study, contrary to some of the results in the literature, low vitamin D levels appeared to occur less in the presence of type 2 diabetes, hypertension, dyslipidaemia and atherosclerotic heart disease. Milk and dairy product consumption did not show a significant effect on vitamin D levels.

### Comparison with existing literature

Our study results indicated that low vitamin D levels are more prominent in women and occur in spring, which is per the literature from Turkey and countries with similar to Turkey in geographical location [4,12,13,17,25].

Concerning the accompanying comorbid situations, contrary to some of the results in the literature, low vitamin D levels appeared to occur less in the presence of hypertension, dyslipidemia and atherosclerotic heart disease in our study [5,6,29,30].

### Limitations

Our study has some limitations. It was a retrospective data analysis. In the whole study population, vitamin D levels might be measured in some patients who did not have a risk of vitamin D deficiency; the questionnaire could be applied to a limited number of patients, the cut-off level of vitamin D taken as 20 ng/mL, according to some guidelines and literature, may lead to overdiagnosis of deficiency.

### Implications for clinical practice

Due to the effects of vitamin D on general and skeletal health, it is crucial to increase awareness among physicians and patients about the vitamin D status of society. Our results are important to inform people to organise their lifestyle habits, leading to more conscious sun exposure and consuming healthy food rich in or enriched with vitamin D.

## Conclusion

Our study indicated that low vitamin D levels in the population of Bursa are more prominent in women, occur in spring, and in situations such as living indoors. These results are meaningful for the population living in both Bursa and Turkey, and they are remarkable for both the Turkish and European populations in the northern European region.

## References

[1] Pilz S, Zittermann A, Trummer C, et al. Vitamin D testing and treatment: a narrative review of current evidence. Endocr Connect. 2019;8:R27–R43. 30650061 10.1530/EC-18-0432 PMC6365669

[2] Society of Endocrinology and Metabolism of Turkey. Osteoporosis and metabolic bone diseases diagnosis and treatment guide. 2019.

[3] Kamboj P, Dwivedi S, Toteja GS. Prevalence of hypovitaminosis D in India & way forward. Indian J Med Res. 2018;148:548–556. 30666982 10.4103/ijmr.IJMR_1807_18 PMC6366270

[4] Hekimsoy Z, Dinç G, Kafesçiler S, et al. Vitamin D status among adults in the Aegean region of Turkey. BMC Public Health. 2010;10:782. 21176241 10.1186/1471-2458-10-782 PMC3022855

[5] Elizondo-Montemayor L, Castillo EC, Rodríguez-López C, et al. Seasonal variation in vitamin D in association with age, inflammatory cytokines, anthropometric parameters, and lifestyle factors in older adults. Mediators Inflamm. 2017;2017:1–14. 10.1155/2017/5719461 PMC5618765 29104377

[6] Walsh JS, Bowles S, Evans AL. Vitamin D in obesity. Curr Opin Endocrinol Diabetes Obes. 2017;24:389–394. 28915134 10.1097/MED.0000000000000371

[7] Ersoy C, Ersoy A. Current approaches in vitamin D treatment. Uludağ Üniversitesi Tıp Fakültesi Dergisi. 2019;45:219–223.

[8] Holick MF, Binkley NC, Bischoff-Ferrari HA, Endocrine Society, et al. Evaluation, treatment, and prevention of vitamin D deficiency: an Endocrine Society clinical practice guideline. J Clin Endocrinol Metab. 2011;96:1911–1930. 21646368 10.1210/jc.2011-0385

[9] American Geriatrics Society Workgroup on Vitamin D Supplementation for Older Adults. Recommendations abstracted from the American Geriatrics Society Consensus Statement on vitamin D for Prevention of Falls and Their Consequences. J Am Geriatr Soc. 2014;62:147–152. 24350602 10.1111/jgs.12631

[10] Holick MF. Vitamin D deficiency. N Engl J Med. 2007;357:266–281. 17634462 10.1056/NEJMra070553

[11] Atli T, Gullu S, Uysal AR, et al. The prevalence of vitamin D deficiency and effects of ultraviolet light on vitamin D levels in elderly Turkish population. Arch Gerontol Geriatr. 2005;40:53–60. 15531023 10.1016/j.archger.2004.05.006

[12] Alagöl F, Shihadeh Y, Boztepe H, et al. Sunlight exposure and vitamin D deficiency in Turkish women. J Endocrinol Invest. 2000;23:173–177. 10803475 10.1007/BF03343702

[13] Guzel R, Kozanoglu E, Guler-Uysal F, et al. Vitamin D status and bone mineral density of veiled and unveiled Turkish women. J Womens Health Gend Based Med. 2001;10:765–770. 11703889 10.1089/15246090152636523

[14] Turkey Statistical Institute, Regional Directorate of Bursa; 2019. Available from: https://www.nufusu.com/il/bursa-nufusu

[15] Maldonado G, Paredes C, Guerrero R, et al. Determination of vitamin D status in a population of ecuadorian subjects. ScientificWorldJournal. 2017;2017:3831275. 28900630 10.1155/2017/3831275 PMC5576417

[16] Forrest KYZ, Stuhldreher WL. Prevalence and correlates of vitamin D deficiency in US adults. Nutr Res. 2011;31:48–54. 21310306 10.1016/j.nutres.2010.12.001

[17] Niculescu DA, Capatina CAM, Dusceac R, et al. Seasonal variation of serum vitamin D levels in Romania. Arch Osteoporos. 2017;12:113. 29230557 10.1007/s11657-017-0407-3

[18] Looker AC, Dawson-Hughes B, Calvo MS, et al. Serum 25-hydroxyvitamin D status of adolescents and adults in two seasonal subpopulations from NHANES III. Bone. 2002;30:771–777. 11996918 10.1016/s8756-3282(02)00692-0

[19] Van der Wielen RP, Löwik MR, van den Berg H, et al. Serum vitamin D concentrations among elderly people in Europe. Lancet. 1995;346:207–210. 7616799 10.1016/s0140-6736(95)91266-5

[20] Hyppönen E, Power C. Hypovitaminosis D in British adults at age 45 y: nationwide cohort study of dietary and lifestyle predictors. Am J Clin Nutr. 2007;85:860–868. 17344510 10.1093/ajcn/85.3.860

[21] Levis S, Gomez A, Jimenez C, et al. Vitamin D deficiency and seasonal variation in an adult South Florida population. J Clin Endocrinol Metab. 2005;90:1557–1562. 15634725 10.1210/jc.2004-0746

[22] Meddeb N, Sahli H, Chahed M, et al. Vitamin D deficiency in Tunisia. Osteoporos Int. 2005;16:180–183. 15197539 10.1007/s00198-004-1658-6

[23] Kull M, Jr, Kallikorm R, Tamm A, et al. Seasonal variance of 25-(OH) vitamin D in the general population of Estonia, a Northern European country. BMC Public Health. 2009;9:22. 19152676 10.1186/1471-2458-9-22 PMC2632995

[24] Bhattoa HP, Nagy E, More C, et al. Prevalence and seasonal variation of hypovitaminosis D and its relationship to bone metabolism in healthy Hungarian men over 50 years of age: the HunMen Study. Osteoporos Int. 2013;24:179–186. 22422303 10.1007/s00198-012-1920-2

[25] Bhattoa HP, Bettembuk P, Ganacharya S, et al. Prevalence and seasonal variation of hypovitaminosis D and its relationship to bone metabolism in community dwelling postmenopausal Hungarian women. Osteoporos Int. 2004;15:447–451. 15205715 10.1007/s00198-003-1566-1

[26] Merlo C, Trummler M, Essig S, et al. Vitamin D deficiency in unselected patients from Swiss primary care: a cross-sectional study in two seasons. PLoS One. 2015;10:e0138613. 26372355 10.1371/journal.pone.0138613 PMC4570784

[27] Gannage-Yared MH, Chemali R, Yaacoub N, et al. Hypovitaminosis D in a sunny country: relation to lifestyle and bone markers. J Bone Miner Res. 2000;15:1856–1862. 10977006 10.1359/jbmr.2000.15.9.1856

[28] Wortsman J, Matsuoka LY, Chen TC, et al. Decreased bioavailability of vitamin D in obesity. Am J Clin Nutr. 2000;72:690–693. 10966885 10.1093/ajcn/72.3.690

[29] Theodoratou E, Tzoulaki I, Zgaga L, et al. Vitamin D and multiple health outcomes: umbrella review of systematic reviews and meta-analyses of observational studies and randomised trials. BMJ. 2014;348:g2035. 24690624 10.1136/bmj.g2035 PMC3972415

[30] Robinson-Cohen C, Hoofnagle AN, Ix JH, et al. Racial differences in the association of serum 25-hydroxyvitamin D concentration with coronary heart disease events. JAMA. 2013;310:179–188. 23839752 10.1001/jama.2013.7228 PMC4150653

